# Exome-Based Genomic Markers Could Improve Prediction of Checkpoint Inhibitor Efficacy Independently of Tumor Type

**DOI:** 10.3390/ijms24087592

**Published:** 2023-04-20

**Authors:** Lorraine Dalens, Julie Lecuelle, Laure Favier, Cléa Fraisse, Aurélie Lagrange, Courèche Kaderbhai, Romain Boidot, Sandy Chevrier, Hugo Mananet, Valentin Derangère, Caroline Truntzer, François Ghiringhelli

**Affiliations:** 1 Department of Medical Oncology, Georges François Leclerc Cancer Center—UNICANCER, 21000 Dijon, France; ldalens@cgfl.fr (L.D.); lfavier@cgfl.fr (L.F.); cfraisse@cgfl.fr (C.F.); alagrange@cgfl.fr (A.L.); cgkaderbhai@cgfl.fr (C.K.); 2 UFR des Sciences de Santé, University of Burgundy-Franche-Comté, 21000 Dijon, France; jlecuelle@cgfl.fr (J.L.); hmananet@cgfl.fr (H.M.); vderangere@cgfl.fr (V.D.); ctruntzer@cgfl.fr (C.T.); 3 Platform of Transfer in Biological Oncology, Georges-Francois Leclerc Cancer Center—UNICANCER, 21000 Dijon, France; 4 UMR INSERM 1231, 21000 Dijon, France; 5 Genomic and Immunotherapy Medical Institute, Dijon University Hospital, 21000 Dijon, France; 6 Department of Biopathology, Georges François Leclerc Cancer Center—UNICANCER, 21000 Dijon, France; rboidot@cgfl.fr (R.B.); schevrier@cgfl.fr (S.C.)

**Keywords:** biomarkers, TMB, TCR, exome sequencing, immunotherapy

## Abstract

Immune checkpoint inhibitors (ICIs) have improved the care of patients in multiple cancer types. However, PD-L1 status, high Tumor Mutational Burden (TMB), and mismatch repair deficiency are the only validated biomarkers of efficacy for ICIs. These markers remain imperfect, and new predictive markers represent an unmet medical need. Whole-exome sequencing was carried out on 154 metastatic or locally advanced cancers from different tumor types treated by immunotherapy. Clinical and genomic features were investigated using Cox regression models to explore their capacity to predict progression-free survival (PFS). The cohort was split into training and validation sets to assess validity of observations. Two predictive models were estimated using clinical and exome-derived variables, respectively. Stage at diagnosis, surgery before immunotherapy, number of lines before immunotherapy, pleuroperitoneal, bone or lung metastasis, and immune-related toxicity were selected to generate a clinical score. *KRAS* mutations, TMB, TCR clonality, and Shannon entropy were retained to generate an exome-derived score. The addition of the exome-derived score improved the prediction of prognosis compared with the clinical score alone. Exome-derived variables could be used to predict responses to ICI independently of tumor type and might be of value in improving patient selection for ICI therapy.

## 1. Introduction

Antitumor immune response relies predominantly on CD8 cytotoxic T lymphocytes. Tumor cells express specific antigens that can activate the adaptative system through antigen recognition by the T-cell receptor (TCR) [1]. However, tumor cells have the ability to develop mechanisms of immune evasion, which favors the development of clinically detectable cancers [2]. One well-known mechanism is the expression of immune checkpoints, such as CTLA-4 (cytotoxic T lymphocyte–associated protein 4) and PD-1 (programmed cell death protein-1), two major proteins with immunoregulatory functions. PD-1 is a marker of exhausted function in CD8 T-cells. Exhausted T cells progressively lose their capacity to produce cytokines and kill tumor cells. Under physiological conditions, immune checkpoints and CD8 exhaustion are involved in the regulation of immune responses against pathogens, as well as in autoimmunity [3]. In the context of cancer, the tumor promotes the differentiation of CD8 T-cells into exhausted T-cells that express checkpoint inhibitors and fail to induce effective antitumor immune response. Checkpoint blockade has progressively emerged as a promising way to restore exhausted T-cell activity and, therefore, re-enhance the antitumor immune response.

In 2011, the anti-CTLA-4 drug ipilimumab was the first immune checkpoint inhibitor (ICI) to be approved by the Food and Drug Administration (FDA) for melanoma [4]. Different molecules targeting other checkpoints such as PD-1, PD-L1 (programmed death-ligand 1) or LAG-3 (lymphocyte-activation gene 3) have rapidly been developed since, revolutionizing the treatment of a wide array of cancer types. After melanoma, ICIs alone or in association with chemotherapies or antiangiogenic therapies also demonstrated a significant benefit in various tumor types, such as non-small-cell lung cancer (NSCLC) [5,6,7], renal cell carcinoma [8,9,10], squamous cell carcinoma of the head and neck [11], small-cell lung cancer [12], urothelial carcinoma [13], endometrial carcinoma [14], cervical carcinoma [15], mesothelioma [16], hepatocellular carcinoma [17], esophageal carcinoma [18], gastric adenocarcinoma [19], and microsatellite instability (MSI) cancers [20].

Novel patterns of unknown response with cytotoxic chemotherapies or targeted agents have been experienced, with potentially durable responses that can be maintained even years after treatment discontinuation [21]. Unfortunately, despite this success, resistance to ICIs restricts the number of patients who yield a durable response. There is thus a need to identify predictive biomarkers to select the patients most likely to respond to ICIs. 

Currently, PDL-1 tumor expression [22] and MSI [20] are the only biomarkers frequently used in routine clinical practice. In 2020, the FDA approved pembrolizumab for adults and children with high Tumor Mutational Burden (TMB), based on the results of the KEYNOTE 158 trial [23]. This phase 2 study showed that patients with TMB-high status (≥10 mutations per megabase) have a better chance of yielding benefit from pembrolizumab monotherapy. However, using TMB alone may be insufficient, and the origin of high TMB, as well as the tumor type, should be considered before using TMB [24]. In mismatch-repair–proficient tumors, TMB-high status was associated with improved survival in a limited subgroup of patients with specific tumor types, including head and neck cancer, NSCLC, and melanoma. Conversely, many patients yield a benefit from ICI despite TMB-low status [25]. Precision medicine is an emerging strategy to improve access to target therapies and includes an extension of indications based on genomic analysis of tumors by analyzing multiple genomic biomarkers associated with ICI response. In many cancer types, genomic mutations are targetable by small inhibitory molecules, leading to a high response rate and better outcome compared with chemotherapies. Recent trials demonstrated the feasibility and the relevance of large genomic testing in order to improve patients outcome [26].

In this retrospective study, we analyzed data derived from exome sequencing performed in the context of the EXOMA 1 and 2 trials [26] in patients treated with ICIs for metastatic solid cancers. The objective of the present study was to identify genomic biomarkers that predict response to immunotherapy and to generate a genomic prediction score for response to ICIs.

## 2. Results

### 2.1. Patient Characteristics 

Among 1234 patients included in the EXOMA 1 and 2 trials, 154 patients with advanced or metastatic solid cancer were included in this retrospective analysis. These 154 patients were all treated with at least one injection of ICI, given as treatment for their advanced or metastatic disease at our center, and had exome sequencing between 2015 and 2020. Complete sequencing data with checked quality control were available for all 154 patients. Among them, we had blood and tumor tissue for 96 (62.3%) patients and tumor tissue only for 58 (37.7%) patients. 

The median age was 62 years (interquartile range (IQR) = (54, 68)). Fifty-five (36%) patients had NSCLC, nineteen (12%) had colorectal cancer, and thirteen (8%) had breast cancer. The 67 (44%) remaining patients were grouped in a category named “other cancers”, comprised gastric, esophageal, pancreatic, biliary tract, anal, upper aerodigestive tract, parotid, kidney, bladder, upper urinary tract, prostatic, ovarian, cervical, endometrial, soft tissue, skin, adrenal and choroidal cancers, and cancer of unknown primary. Sixty-seven patients (44%) underwent surgery before immunotherapy, mostly with curative intent. Most patients (77%) received immunotherapy (anti-PD1 or anti-PD-L1) as monotherapy. Two-thirds of patients received immunotherapy in the first or second line (*n* = 101; 66%) and 53 (34%) patients in the third line or more.

In the overall population, 22% of patients were considered as responders (complete or partial response) and 78% experienced stable or progressive disease (non-responders). 

No significant difference was observed between the training (*n* = 101) and validation cohorts (*n* = 53). The detailed clinical characteristics of the patients are described in Table 1.

Median PFS survival under immunotherapy was 2.8 months (95% confidence interval (CI) [2.6, 4.4]) for the entire cohort, with PFSs of 2.7 [2.5, 3.4], 8.4 [5.2, not reached (NR)], 2.6 [1.2, NR], and 2.8 [2, 5.4] months, respectively, for NSCLC, colorectal cancer, breast cancer, and other cancers. Median PFS in the training cohort was 2.7 [2.5, 4.5] months, and it was 3.2 [2.6, 5.4] months in the validation cohort. 

**Figure 1:**
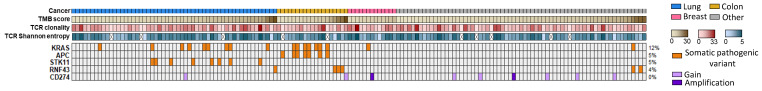
Genomic landscape of genes associated with response to immune checkpoint inhibitors. Tumor samples are sorted by cancer type and ascending order for TMB score. TMB: Tumor Mutational Burden, TCR: T-Cell Receptor.

Genomic structural analysis determined TMB, the number of neoantigens, MSI score, CNV signatures, and TCR and BCR clonality. For TCR clonality, 734 clones were identified in the whole cohort, with 159 expressed at least by 2 patients (Supplementary Figure S1A). Two hundred eighty-seven BCR clones were identified with two hundred seventy-six clones expressed by only one patient and eleven by two patients (Supplementary Figure S1B). In the whole cohort, 20 patients (13%) had TMB-high status (using the classical cut-off of 10 mutations per Mb), 8 (5.2%) had MSI, and 18 patients (12%) presented a *KRAS* mutation—especially in NSCLC and colorectal cancers (Figure 1). No significant difference was observed between training and validation cohorts (Table 2). Note that no patient had pathogenic or likely pathogenic variants for *KEAP1* and *RSPO3*.

### 2.2. TMB Score and Type of Cancer

**Figure 2:**
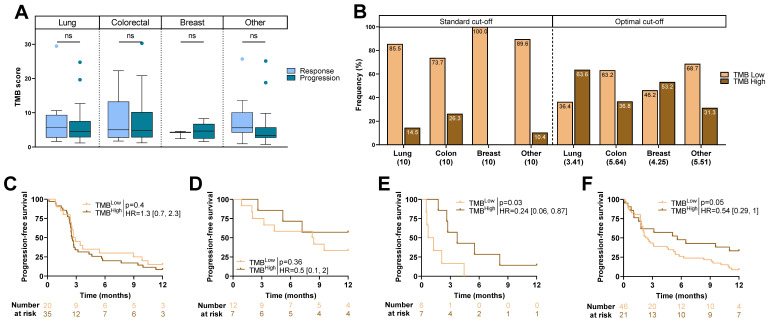
TMB score and outcome: (**A**) Boxplots showing TMB score according to RECIST criteria for each cancer. (**B**) Barplots representing frequency of TMB^High^ and TMB^Low^ patients dichotomized according to the standard cut-off and optimal cut-off for each cancer (cut-off in brackets). (**C**–**F**) Kaplan–Meier curves with patients stratified according to TMB status (optimal cut-off) for progression-free survival for (**C**) non-small-cell lung cancer, (**D**) colorectal cancer, (**E**) breast cancer, and (**F**) other cancers. ns: not significant.

To further investigate the role of TMB, the TMB score was analyzed according to cancer type. Continuous TMB score was not associated with RECIST status (Figure 2A). Using the standard cut-off, high TMB was not observed in breast cancer but was present in 15% of patients with NSCLC, 26% of patients with colorectal cancer, and 10% in other cancers (Figure 2B). Moreover, high TMB status was not associated with PFS in any cancer type (results not shown). The optimal TMB cut-off to distinguish patients according to PFS changed according to the tumor type, ranging from 3.41 to 5.64 (R library maxstat). Using the optimal cut-off, high TMB was observed in 64% of patients with NSCLC, 37% of patients with colorectal cancer, 54% of patients with breast cancer, and 31% in other cancers (Figure 2B). Subgroup analysis showed that using the optimal cut-off, high TMB was only significantly associated with better PFS in breast cancer (HR = 0.24 [0.06, 0.87]; *p* = 0.03), and a trend was observed in other cancers (HR = 0.54 [0.29, 1]; *p* = 0.05). TMB was not related to outcome in colorectal cancer (Figure 2C–F). 

### 2.3. Association between Clinical Variables and Outcome

In the overall population, patients treated with ICIs in the first or second line and patients who experienced immune-related toxicity had a higher response rate (Supplementary Table S1). 

**Figure 3:**
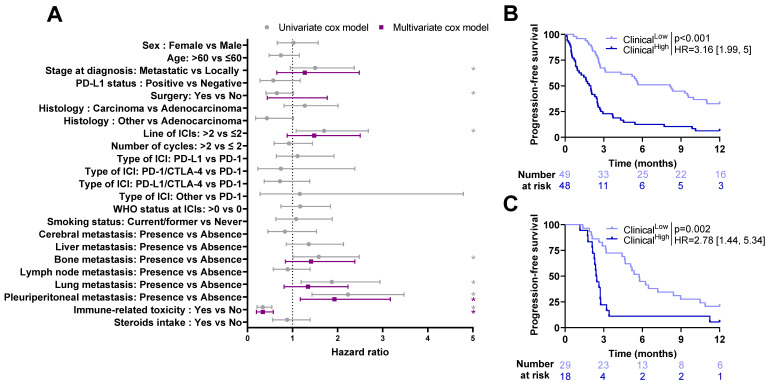
Association between progression-free survival and clinical variables: (**A**) Forest plots representing hazard ratios and confidence intervals for univariate (gray) and multivariate (purple) Cox models for progression-free survival estimated using clinical variables in the training cohort. *: Log-rank test *p*-value ≤ 0.1. (**B**,**C**) Kaplan–Meier curves with patients stratified according to the clinical model dichotomized by training median (High vs. Low) for progression-free survival for training cohort (**B**) and validation cohort (**C**). ICI: Immune Checkpoint Inhibitor; PD-L1: Programmed Death-Ligand 1; PD-1; Programmed cell Death protein-1; CTLA-4: Cytotoxic T Lymphocyte-Associated protein 4; WHO: World Health Organization.

In the training cohort, patients with local stage at diagnosis, surgery before immunotherapy, ICIs in the first or second line of treatment, as well as patients who experienced immune related toxicity and those without bone, lung or pleuroperitoneal metastasis had significantly longer PFS. Among these factors, only immune-related toxicity and presence of pleuroperitoneal metastasis remained significant by multivariate analysis (*p*-value < 0.05) (Figure 3A). 

All variables significantly related to PFS by univariate analysis were selected to estimate a multivariate clinical model to predict patient prognosis. The linear predictor of this model was then used as clinical composite variable and dichotomized (High vs. Low) based on its median estimated in the training cohort. Patients in the “High” group had a significantly poorer PFS (HR = 3.16 [1.99, 5]; *p* < 0.001, Figure 3B). Similar results were observed when applying this score in the validation cohort (HR = 2.78 [1.44, 5.34]; *p* = 0.002, Figure 3C).

### 2.4. Association between Exome-Derived Variables and Outcome

In the overall total population, no significant difference was observed between responders and progressors (Supplementary Table S2). 

**Figure 4:**
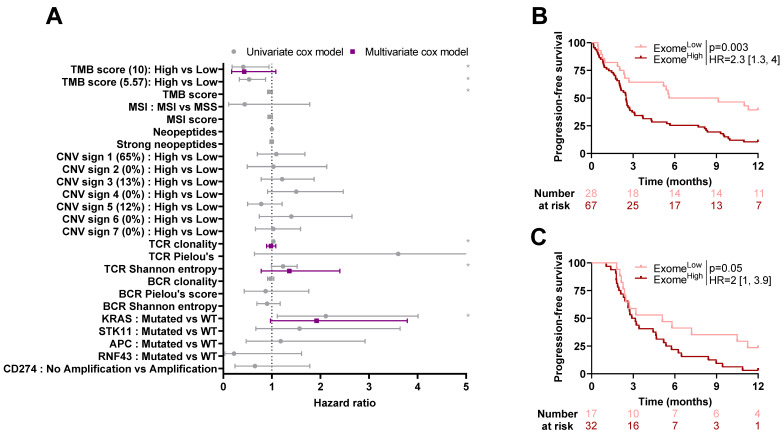
Association between progression-free survival and exome-derived variables: (**A**) Forest plots representing hazard ratios and confidence intervals for univariate (gray) and multivariate (purple) Cox models for progression-free survival estimated using exome-derived variables in the training cohort. *: Log-rank test *p*-value ≤ 0.1. (**B**,**C**) Kaplan–Meier curves with patients stratified according to the exome model dichotomized by optimal cut-off (High vs. Low) for progression-free survival for the training cohort (**B**) and validation cohort (**C**). TMB: Tumor Mutational Burden; MSI: Microsatellite Instability; MSS: Microsatellite Stable; CNV: Copy Number Variant; TCR: T-Cell Receptor; BCR: B-cell Receptor; WT: Wild-Type.

By univariate analysis, low TMB (using the classical cut-off of 10 mutations per Mb), high TCR clonality, high TCR Shannon entropy, and presence of *KRAS* mutation were associated with poorer PFS for patients in the training cohort (Figure 4A). No variable remained significant by multivariate analysis. 

An exome-derived model was estimated including variables that were significant by univariate analysis. The linear predictor of this model was then used as an exome-derived composite variable and dichotomized (High vs. Low) based on optimal cut-off estimated in the training cohort through maximally selected rank statistic. This variable dichotomized patients of the training cohort into two groups with different PFS (High vs. Low: HR = 2.3 [1.3, 3.4]; *p* = 0.003, Figure 4B). This score remained significant when applied in the validation cohort after re-estimating a cut-off proper to this cohort (HR = 2 [1, 3.9]; *p* = 0.05, Figure 4C). 

### 2.5. Exome-Derived Variables Add Predictive Power on Top of Clinical Variables

A combined model was estimated with clinical and exome-derived variables to test the capacity of a combined model to improve predictive power. The linear predictor of this model was then used as combined composite variable and dichotomized (High vs. Low) based on its median estimated in the training cohort.

**Figure 5:**
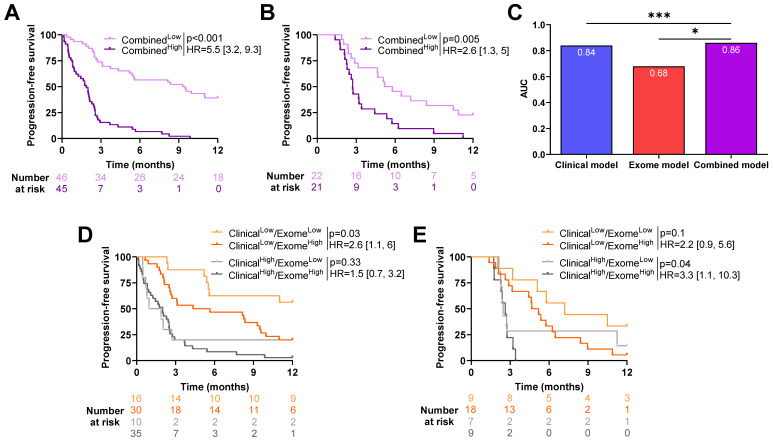
Association between progression-free survival and clinical and exome-derived variables. Kaplan–Meier curves with patients stratified according to dichotomized linear predictors obtained from the combined model for progression-free survival in (**A**) training cohort and (**B**) validation cohort. (**C**) Barplots of time-dependent AUC (Area Under the Curve) for clinical, exome, and combined (clinical and exome) models for progression-free survival. * *p* < 0.05, *** *p* < 0.001. Kaplan–Meier curves with patients stratified according to clinical and exome models for progression-free survival for the (**D**) training cohort and (**E**) validation cohort.

In both cohorts, patients in the Low group had better PFS (HR = 0.18 [0.11, 0.31]; *p* < 0.001 in the training cohort and HR = 0.39 [0.2, 0.75]; *p* = 0.005 in the validation cohort, Figure 5A,B). A comparison of the models using the likelihood ratio test showed that the exome-derived model improved the predictive power of the clinical model (*p*-value = 0.02, Figure 5C).

Four groups were then created using composite clinical and exome-derived variables. Patients classified as Clinical^Low^/Exome-derived^Low^ had significantly better PFS than patients classified as Clinical^Low^/Exome-derived^High^ in the training cohort (HR = 0.39 [0.17, 0.91]; *p* = 0.03, Figure 5D). For patients classified as Clinical^High^, exome-derived status had no impact on survival (HR = 0.68 [0.31, 0.47]; *p* = 0.33). 

In the validation cohort, the exome-derived variable allowed to further discriminate patients between high and low risk for patients with Clinical^High^ status; in fact, patients classified as Exome-derived^Low^ had a significantly better PFS (HR = 3.3 [1.1, 10.3]; *p* = 0.04). This was not significant for patients classified as Clinical^Low^ (HR = 0.46 [0.18, 1.18]; *p* = 0.1, Figure 5E).

These observations highlight the contribution of the exome-derived variable to clinical variables.

## 3. Discussion

Over the last decade, ICIs have revolutionized the management of cancer, requiring a rethinking of the treatment strategies that have been used for many years. However, ICIs only benefit a small proportion of patients, and to date, no predictive biomarker has been shown to be sufficiently robust to exhaustively select patients likely to respond to immunotherapy. In this study, we analyzed clinical variables and data derived from exome analysis to predict PFS under ICIs in all types of cancers, independently of location and histologic type. 

Analysis of clinical variables revealed that PFS is longer when ICIs are administered in the first or second line of treatment, as well as when patients do not have bone, lung or pleuroperitoneal metastasis. Patients with local stage at diagnosis, surgery before immunotherapy, and those who present immune-related toxicity also have better PFS. Regarding exome-derived variables, high TMB, low TCR clonality, low Shannon entropy, and wild-type KRAS status were found to be associated with longer PFS. As previously shown by Litchfield et al., concerning efficacy in a large meta-analysis involving seven different tumor subtypes [27], TMB stands out as a major predictive factor for immunotherapy. However, those results are not confirmed by the analysis of Rousseau et al. [24], which questions the FDA approval for ICI on the basis of high TMB, using a single-center cohort of 1661 patients treated by ICI. In their analysis, they observed that high TMB was only associated with better survival in the case of MSI- or *POLD (POLE or POLD1)*-mutated tumors, or in cancers highly related to environmental carcinogens (head and neck, lung, and melanoma). 

To perform MSI assessment, we used MSIsensor software (v3.0.4), which generates an MSI score using data from the exome. With a cut-off of 20, this score demonstrated its reliability for the identification of MSI tumors [28]. Only eight patients had MSI in our cohort. Among these, only seven patients had an available RECIST response, and three had complete or partial responses (43%), while four had stable or progressive diseases (57%). This is consistent with the response rate found in the KEYNOTE 158 [20] (objective response rate of 34%). MSI status did not stand out as a predictive factor for ICI efficacy in our study. With only 5.2% of patients having MSI, we probably did not have sufficient power to show a statistically significant difference.

In our study, *KRAS* mutations were associated with shorter PFS. Generally, *KRAS*-activated mutations are considered as a pejorative prognostic factor [29]. In colorectal cancer, *KRAS* mutation is also widely associated with poor prognosis [30]. Similar results have been observed in NSCLC cancer [31]. In contrast, previous studies provided evidence that *KRAS* mutation was associated with a better response to immunotherapy, especially in NSCLC. In the Checkmate-057 study, *KRAS* wild-type NSCLC did not benefit from nivolumab (versus docetaxel) in the second line [6]. Our data are in opposition with these results, and additional data are warranted to better understand the influence of *RAS* on the efficacy of immunotherapy.

We show here that a lower number of TCR clones and low Shannon entropy were associated with better PFS, suggesting that restricted diversity is predictive of a better immune response than tumors with polyclonal nonspecific T-cell infiltration. This is consistent with a previous study by Valpione et al. [32], where they showed that while high TCR diversity seemed to be a prognostic factor in cancer patients, high TCR clonality (implying lower diversity) was a predictive factor for response to ICIs. In a previous publication, using another dataset of patients with NSCLC treated with nivolumab in the second line, our group reported that restriction in the number of TCR clones was also associated with good PFS [33].

Our study has some limitations, notably the small number of patients, the single-center design, and the heterogeneity of patients with various tumor types, treatments, and lines of therapy.

## 4. Materials and Methods

Patients with locally advanced unresectable or metastatic solid cancer treated with ICIs at the Georges-François Leclerc Cancer Center (Dijon, France) who had exome sequencing were included in this retrospective single-center study. All of them were prospectively included in the EXOMA trial (NCT02840604 and NCT04614480). The exome sequencing was performed prospectively according to the EXOMA trial protocol.

Genomic analyses were performed at the Georges-Francois Leclerc Cancer Center in the Genomic and Immunotherapy Medical Institute, Dijon, France. All patients provided signed informed consent for the trial and genomic analysis. After informed consent, patients had a consultation with a genetic counsellor before the constitutional exome analysis. 

The dedicated analysis for the purposes of the present study was performed retrospectively and was not the main purpose of the original EXOMA trial.

Patient and tumor characteristics were collected, namely sex, age, WHO Performance Status (PS), smoking history, primary organ, histologic type, date of diagnosis, stage at diagnosis, sites of metastasis, medical treatments, surgery of the primary cancer or the metastasis performed before ICI administration, best response to ICIs, immune-related toxicities, and steroid intake during ICI therapy. The best response assessment was based on computed tomography (CT) scans using the RECIST 1.1 criteria. In case of unconfirmed progressive disease, reassessment was performed four to eight weeks later. Patients were considered as responders if they experienced complete response (CR) or partial response (PR) to ICI. They were classified as progressors if they had a stable disease (SD) or progressive disease (PD).

The database was registered with the National French Commission on Informatics and Liberty (CNIL). The study was conducted in accordance with French legislation and the Declaration of Helsinki, with approval from CPP and ANSM as required. 

### 4.1. Sample Selection

After obtaining written informed consent for the EXOMA study, physicians selected an archival tumor sample dating from less than one year (primary or metastasis) for genomic analysis. At the physician’s discretion, a new tumor biopsy could be proposed to the patient. Tumor cellularity was assessed by a senior pathologist on hematoxylin and eosin slides from the same biopsy core as that used for nucleic acid extraction and molecular analysis.

### 4.2. DNA Isolation 

DNA was isolated from archival tumor tissue using the Maxwell 16 FFPE Plus LEV DNA Purification kit (Promega, Madison, WI, USA). DNA from whole blood (germline DNA) was isolated using the Maxwell 16 Blood DNA Purification Kit (Promega) according to the manufacturer’s instructions. The quantity of extracted genomic DNA was assessed by a fluorimetric method with a Qubit device. 

### 4.3. Whole-Exome Capture and Sequencing

Two hundred ng of genomic DNA was used for library preparation, using the Agilent SureSelectXT reagent kit (Agilent Technologies, Santa Clara, CA, USA). The totality of the enriched library was used in the hybridization and captured with the SureSelect All Exon v5 or v6 (Agilent Technologies) baits. Following hybridization, the captured libraries were purified according to the manufacturer’s recommendations and amplified by polymerase chain reaction (12 cycles). Normalized libraries were pooled, and DNA was sequenced on an Illumina NextSeq500 device using 2 × 111 bp paired-end reads and multiplexed. Tumor and germline DNA sequencing generated mean target coverages of 78× and 90×, respectively, and a mean of more than 90% of the target sequence was covered with a read depth of at least 10× for somatic DNA.

### 4.4. Exome Analysis Pipeline

As paired normal-tumor samples were not available for the whole cohort, only tumor samples were considered in this analysis. 

Reads in the FASTQ format were aligned to the reference human genome GRCh37 using the Burrows–Wheeler aligner (BWA v0.7.17). Local realignment was performed using the Genome Analysis Toolkit (GATK v4.1.3.0). Duplicate reads were removed using Picard v.2.5. Single-nucleotide variants (SNVs) were detected using Mutect2 (GATK v 4.1.3.0) variant caller.

TMB was calculated using the number of significant SNVs (with Untranslated Transcribed Region, synonyms, introns, and intergenic SNVs filtered out) divided by the number of megabases covered at a defined level.

To identify tumor-specific mutant peptides, pVAC-Seq (personalized Variant Antigens by Cancer Sequencing) [34] was used (pVACtools v 1.5.4). This computational workflow compares and differentiates the epitopes found in normal cells against the neoepitopes specifically present in tumor cells to predict neoantigens. pVAC-Seq is based on HLA typing obtained by HLAminer (v1.4) [35]. 

The microsatellite instability (MSI) score was computed using MSIsensor (v0.5) [36]. Copy number alterations were inferred using SuperFreq algorithm [37]. Copy number variant signatures were then inferred following the methodology of Macintyre et al. [38]. With this method, the copy number profile of each patient was reconstructed based on the weighted combination of 7 signatures.

Presence and quantitation of T-cell receptor (TCR) and B-cell receptor (BCR) clones were determined using the MixCR software (v3.0.4) [39], available at http://mixcr.milaboratory.com/ (accessed on 1 March 2023). In the present analysis, clonotypes were assembled based on CDR3 sequence only, making it possible to estimate the frequency and clonality of T and B cells at the tumor site. Population diversity of TCR or BCR repertoires can be quantitatively expressed by two separate factors: diversity (i.e., the number of unique elements in a population) and clonality (i.e., the frequency distribution of those elements). Diversity of each sample was calculated using the Shannon entropy index, which takes into account both sample richness and the degree of unevenness in the frequencies of CDR3 sequence, thus meaning that the higher the Shannon entropy index, the more diverse the CDR3 clone distribution [40]. Clonal evenness of each sample was calculated using Pielou’s index, which equals the ratio between the Shannon entropy index and the maximization of the diversity distribution of the CDR3 sequence. Therefore, a Pielou’s index close to 1 represents a maximally diverse population, with each CDR3 clone having a frequency close to 1 [41].

### 4.5. Analysis of Somatic Mutations

We limited our analysis to a short list of genes reported to be associated with response to ICI, namely *KEAP1*, *STK11*, *CD274*, *RNF43*, *RSPO3*, *KRAS*, and *APC* [42,43,44,45].

According to the literature and knowledge databases, each selected variant was classified as “pathogenic”, “likely pathogenic”, “unknown pathogenicity”, or “benign”. For each detected and annotated variant, we retained for interpretation only variants classed as pathogenic or likely pathogenic. Unknown variants were retained when present in somatic analysis only and located in a critical domain of the protein. Each therapeutic proposal was then classified using the ESCAT recommendations [46]. 

### 4.6. Statistical Analysis

Given the small number of patients in each cancer type, all cancer types were combined into a global cohort. This cohort was randomly split into two groups, two thirds of the patients in a training set (*n* = 101) and one third of the patient in an unseen validation set (*n* = 53).

Patient characteristics are described as median and interquartile range (IQR) for continuous variables and as number and percentage (%) for qualitative variables. 

All characteristics were compared by cohort (training or validation) or by group of RECIST criteria using the Chi-2 or Fisher’s exact test for qualitative variables, or the Wilcoxon test for continuous variables, as appropriate. *p*-values were adjusted using Benjamini–Hochberg FDR correction, and adjusted *p*-values < 0.05 were considered significant [47]. 

Progression-free survival (PFS) was calculated as the time from the start of immunotherapy until disease progression and was censored at five years. 

Survival analysis was performed using the survival R library. The prognostic value of the different variables was tested using univariate and multivariate Cox models for PFS in the training cohort. Survival probabilities were estimated using the Kaplan–Meier method, and survival curves were compared using the log-rank test. Variables with unadjusted *p*-values < 0.10 in univariate analysis were selected for multivariate analysis. TMB score was dichotomized based on the cut-off value determined using the maximally selected rank statistics from the maxstat R library [48]. CNV signatures were dichotomized based on their median computed on the training and validation cohorts, respectively.

Three multivariate prognostic models were estimated, one including clinical variables only, one including exome-derived variables, and a last one combining clinical and exome-derived variables. In each case, all variables associated with PFS by univariate Cox models with a *p*-value < 0.1 were included in the multivariate Cox model. For each model, a composite score was estimated based on the corresponding linear predictor of the Cox model. These scores were then dichotomized (High vs. Low) based on the cut-off value determined using either the maximally selected rank statistics or the median. 

Nested models were compared using the likelihood ratio test (LRT) and the Area Under the Curve (AUC).

Data were analyzed using R (version 4.0.3) statistical software (http://www.R-project.org/, accessed on 1 March 2023), and graphics were prepared with Prism 9 (GraphPad, San Diego, CA, USA).

## 5. Conclusions

In conclusion, our study showed that WES could provide useful information to predict response to ICI independently of tumor type. It supports the concept that in a cancer-agonist manner, TCR diversity could be used in combination with TMB to improve patient prognostic prediction.

## Figures and Tables

**Table 1:** Clinical characteristics for the study population (whole cohort and training and validation cohorts).

Variables	Whole Cohort N = 154	Training Cohort N = 101	Validation Cohort N = 53	*p*-Value	Adjusted *p*-Value
Sex				0.9	>0.9
Male	80 (52%)	53 (52%)	27 (51%)		
Female	74 (48%)	48 (48%)	26 (49%)		
Age, years	62 (54, 68)	62 (55, 68)	61 (54, 67)	0.6	0.9
≤60	66 (43%)	42 (42%)	25 (47%)	0.5	0.8
>60	87 (57%)	59 (58%)	28 (53%)		
NA	1	1	0		
Stage at diagnosis				0.044	0.4
Local	70 (45%)	40 (40%)	30 (57%)		
Metastatic	84 (55%)	61 (60%)	23 (43%)		
PDL1 status				>0.9	>0.9
Positive	18 (30%)	13 (30%)	5 (29%)		
Negative	43 (70%)	31 (70%)	12 (71%)		
NA	93	57	36		
Surgery				0.2	0.7
No	87 (56%)	61 (60%)	26 (49%)		
Yes	67 (44%)	40 (40%)	27 (51%)		
Type of surgery				0.2	0.7
Curative	60 (90%)	34 (85%)	26 (96%)		
Palliative	7 (10%)	6 (15%)	1 (3.7%)		
Histology				>0.9	>0.9
Adenocarcinoma	81 (53%)	54 (53%)	27 (51%)		
Carcinoma	55 (36%)	35 (35%)	20 (38%)		
Other	18 (12%)	12 (12%)	6 (11%)		
Line of ICI				0.5	0.8
≤2	101 (66%)	68 (67%)	33 (62%)		
>2	53 (34%)	33 (33%)	20 (38%)		
Number of cycles				0.4	0.8
≤2	59 (38%)	41 (41%)	18 (34%)		
>2	95 (62%)	60 (59%)	35 (66%)		
Type of ICI				0.5	0.8
PD-1	93 (60%)	61 (60%)	32 (60%)		
PD-L1	26 (17%)	20 (20%)	6 (11%)		
PD-1/CTLA-4	4 (2.6%)	3 (3.0%)	1 (1.9%)		
PD-L1/CTLA-4	27 (18%)	15 (15%)	12 (23%)		
Other	4 (2.6%)	2 (2.0%)	2 (3.8%)		
WHO status at ICI				>0.9	>0.9
0	55 (36%)	36 (36%)	19 (36%)		
>0	98 (64%)	64 (64%)	34 (64%)		
NA	1	1	0		
Smoking status				0.9	>0.9
Never smoker	35 (29%)	22 (28%)	13 (30%)		
Smoker	87 (71%)	56 (72%)	31 (70%)		
NA	32	23	9		
Cerebral metastasis	25 (16%)	17 (17%)	8 (15%)	0.8	>0.9
NA	1	0	1		
Liver metastasis	47 (31%)	35 (35%)	12 (23%)	0.14	0.7
NA	1	0	1		
Bone metastasis	51 (33%)	35 (35%)	16 (31%)	0.6	0.9
NA	1	0	1		
Lymph node metastasis	106 (70%)	65 (64%)	41 (80%)	0.042	0.4
NA	2	0	2		
Lung metastasis	60 (42%)	38 (39%)	22 (47%)	0.4	0.8
NA	10	4	6		
Pleuro-peritoneal metastasis	58 (38%)	42 (42%)	16 (31%)	0.2	0.7
NA	1	0	1		
Toxicity	66 (43%)	41 (41%)	25 (47%)	0.4	0.8
Use of corticosteroids	51 (33%)	39 (39%)	12 (23%)	0.045	0.4
RECIST				0.3	0.8
Progression	115 (78%)	71 (76%)	44 (83%)		
Response	32 (22%)	23 (24%)	9 (17%)		
NA	7	7	0		
Cancer type				0.2	0.7
Breast	13 (8%)	12 (12%)	1 (1.9%)		
Colon	19 (12%)	12 (12%)	7 (13%)		
Lung	55 (36%)	36 (36%)	19 (36%)		
Other	67 (44%)	41 (41%)	26 (49%)		

Continuous variables are described by median values and interquartile range (IQR). Categorical variables are described by number of observation and percentages (%). NA: Not Available; ICI: Immune Checkpoint Inhibitor; PD-1: Programmed cell Death protein 1; PD-L1: Programmed Death-Ligand 1; CTLA-4: Cytotoxic T Lymphocyte-Associated protein 4; WHO: World Health Organization.

**Table 2:** Exome-derived variables for the whole cohort and the training and validation cohorts.

Variables	Whole Cohort N = 154	Training Cohort N = 101	Validation Cohort N = 53	*p*-Value	Adjusted *p*-Value
TMB status				0.3	>0.9
Low	134 (87%)	90 (89%)	44 (83%)		
High	20 (13%)	11 (11%)	9 (17%)		
TMB score	4.5 (2.8, 7.1)	4.5 (3.1, 6.1)	4.3 (2.6, 8.0)	>0.9	>0.9
MSI status				0.4	>0.9
MSS	146 (95%)	97 (96%)	49 (92%)		
MSI	8 (5.2%)	4 (4.0%)	4 (7.5%)		
MSI score	1.3 (1.0, 1.9)	1.4 (1.0, 1.9)	1.2 (0.9, 1.7)	0.4	>0.9
TCR clonality	7 (4, 12)	7 (4, 12)	7 (4, 10)	0.5	>0.9
BCR clonality	0.00 (0.00, 2.00)	0.00 (0.00, 2.00)	0.00 (0.00, 2.00)	>0.9	>0.9
NA	2	2	0		
Neopeptides	12 (7, 28)	13 (6, 36)	11 (7, 23)	0.6	>0.9
NA	3	2	1		
Strong neopeptides	2.0 (1.0, 5.0)	2.0 (1.0, 5.0)	2.0 (1.0, 3.2)	0.4	>0.9
NA	3	2	1		
CNV signature 1	68 (53, 79)	65 (51, 76)	72 (61, 81)	0.2	>0.9
Cut-off = 65.3%, 71.7% Low		50 (50%)	26 (50%)	>0.9	>0.9
High		50 (50%)	26 (50%)		
NA	2	1	1		
CNV signature 2	0.00 (0.00, 0.00)	0.00 (0.00, 0.00)	0.00 (0.00, 0.00)	0.4	>0.9
Cut-off = 0%, 0%, Low		90 (90%)	49 (94%)	0.5	>0.9
High		10 (10%)	3 (5.8%)		
NA	2	1	1		
CNV signature 3	13 (10, 19)	13 (10, 19)	13 (10, 20)	>0.9	>0.9
Cut-off = 13.2%, 13%, Low		50 (50%)	26 (50%)	>0.9	>0.9
High		50 (50%)	26 (50%)		
NA	2	1	1		
CNV signature 4	0.00 (0.00, 0.00)	0.00 (0.00, 0.00)	0.00 (0.00, 0.00)	0.7	>0.9
Cut-off = 0%, 0%, Low		77 (77%)	42 (81%)	0.6	>0.9
High		23 (23%)	10 (19%)		
NA	2	1	1		
CNV signature 5	11 (5, 18)	0.12 (0.06, 0.19)	10 (4, 16)	0.12	>0.9
Cut-off = 11.8%, 9.8%, Low		50 (50%)	26 (50%)	>0.9	>0.9
High		50 (50%)	26 (50%)		
NA	2	1	1		
CNV signature 6	0.00 (0.00, 0.00)	0.00 (0.00, 0.00)	0.00 (0.00, 0.00)	>0.9	>0.9
Cut-off = 0%, 0%, Low		88 (88%)	46 (88%)	>0.9	>0.9
High		12 (12%)	6 (12%)		
NA	2	1	1		
CNV signature 7	0.00 (0.00, 0.08)	0.00 (0.00, 11)	0.00 (0.00, 4)	0.2	>0.9
Cut-off = 0%, 0%, Low		55 (56%)	34 (65%)	0.2	>0.9
High		44 (44%)	18 (35%)		
NA	3	2	1		
TCR Pielou’s score	0.98 (0.96, 1.00)	0.99 (0.96, 1.00)	0.97 (0.95, 1.00)	0.3	>0.9
NA	10	6	4		
BCR Pielou’s score	1.00 (0.72, 1.00)	0.99 (0.00, 1.00)	1.00 (0.96, 1.00)	0.078	>0.9
NA	84	55	29		
TCR Shannon entropy	2.81 (2.00, 3.54)	2.85 (2.00, 3.55)	2.75 (2.00, 3.32)	0.6	>0.9
NA	10	6	4		
BCR Shannon entropy	1.44 (0.72, 2.00)	1.26 (0.00, 2.30)	1.48 (1.00, 2.00)	0.7	>0.9
NA	84	55	29		
KRAS				0.7	>0.9
WT	136 (88%)	90 (89%)	46 (87%)		
Mutated	18 (12%)	11 (11%)	7 (13%)		
STK11				0.7	>0.9
WT	146 (95%)	95 (94%)	51 (96%)		
Mutated	8 (5.2%)	6 (5.9%)	2 (3.8%)		
APC				>0.9	>0.9
WT	147 (95%)	96 (95%)	51 (96%)		
Mutated	7 (4.5%)	5 (5.0%)	2 (3.8%)		
RNF43				0.4	>0.9
WT	148 (96%)	98 (97%)	50 (94%)		
Mutated	6 (3.9%)	3 (3.0%)	3 (5.7%)		
CD274				0.5	>0.9
Amplification	9 (5.8%)	5 (5.0%)	4 (7.5%)		
No amplification	145 (94%)	96 (95%)	49 (92%)		

Continuous variables are described by median values and interquartile range (IQR). Categorical variables were described by number of observation and percentages (%). TMB: Tumor Mutational Burden; MSI: Microsatellite Instability; MSS: Microsatellite Stable; TCR: T-Cell Receptor; BCR: B-Cell Receptor; CNV: Copy Number Variant.

## Data Availability

Data are available from authors upon reasonable request.

## Supplementary Materials

The supporting information can be downloaded at: https://www.mdpi.com/article/10.3390/ijms24087592/s1.

## Abbreviations

AUC	Area Under the Curve
BCR	B-cell receptor
CNV	Copy Number Variant
CT	Computed Tomography
CTLA-4	Cytotoxic T Lymphocyte–Associated protein 4
CR	Complete Response
FDA	Food and Drug Administration
FDR	False Discovery rate
HR	Hazard Ratio
ICI	Immune Checkpoint Inhibitor
IQR	Interquartile Range
LAG-3	Lymphocyte-Activation Gene 3
LRT	Likelihood Ratio Test
MSI	Microsatellite Instability
MSS	Microsatellite Stable
NS	Not Significant
NSCL	Non-Small-Cell Lung cancer
NR	Not Reached
PD	Progressive Disease
PD1	Programmed cell Death protein-1
PD-L1	Programmed Death-Ligand 1
PFS	Progression-Free Survival
PR	Partial Response
PS	Performance Status
SD	Stable Disease
SNV	Single-Nucleotide Variants
TMB	Tumor Mutational Burden
TCR	T-cell Receptor
WHO	World Health Organization
WT	Wild-Type
