# Supplementary material for: Exome-Based Genomic Markers Could Improve Prediction of Checkpoint Inhibitor Efficacy Independently of Tumor Type
Source: Int J Mol Sci. 2023 Apr 20;24(8):7592. doi: 10.3390/ijms24087592 (PMC10144126; doi:10.3390/ijms24087592)
Supplement: Supplementary file 1 [file ijms-24-07592-s001.zip › Supplementary_Figure S1.pdf]

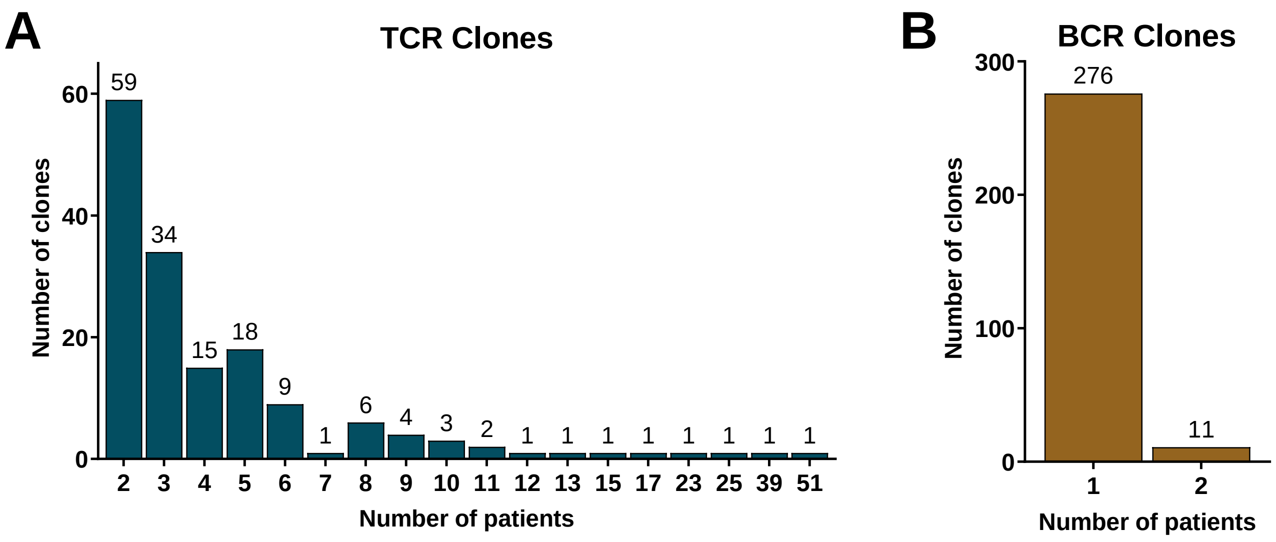

Supplementary Figure S1: Description of TCR and BCR clonality
Barplots representing the number of clones shared by increasing numbers of patients for A) TCR clones and B) BCR clones.
