# Supplementary material for: Exome-Based Genomic Markers Could Improve Prediction of Checkpoint Inhibitor Efficacy Independently of Tumor Type
Source: Int J Mol Sci. 2023 Apr 20;24(8):7592. doi: 10.3390/ijms24087592 (PMC10144126; doi:10.3390/ijms24087592)
Supplement: Supplementary file 1 [file ijms-24-07592-s001.zip › Supplementary_tables.pdf]

Supplementary Table S1: Clinical characteristics for the whole cohort according to RECIST criteria.

Variables	Progression N=115	Response N=32	P-value	Adjusted P-value
Gender			0.7	0.7
Male	60 (52%)	18 (56%)		
Female	55 (48%)	14 (44%)		
Age	61 (56, 67)	66 (58, 71)	0.2	0.5
≤60	50 (43%)	11 (34%)	0.4	0.6
>60	65 (57%)	21 (66%)		
NA	1	0		
Stage at diagnosis			0.4	0.6
Local	53 (46%)	12 (38%)		
Metastatic	62 (54%)	20 (62%)		
PDL1 status			0.11	0.3
Positive	16 (37%)	2 (12%)		
Negative	27 (63%)	14 (88%)		
NA	72	16		
Surgery			0.6	0.7
Yes	66 (57%)	20 (62%)		
No	49 (43%)	12 (38%)		
Type of surgery			0.6	0.7
Curative	44 (90%)	10 (83%)		
Palliative	5 (10%)	2 (17%)		
Histology			0.3	0.5
Adenocarcinoma	60 (52%)	19 (59%)		
Carcinoma	44 (38%)	8 (25%)		
Other	11 (9.6%)	5 (16%)		
Line of ICI			<0.001	0.02
≤2	68 (59%)	29 (91%)		
>2	47 (41%)	3 (9.4%)		
Number of cycles			0.8	0.9
≤2	41 (36%)	12 (38%)		
>2	74 (64%)	20 (62%)		
Type of ICI			0.5	0.6
PD-1	72 (63%)	18 (56%)		
PD-L1	19 (17%)	4 (12%)		
PD-1/CTLA-4	2 (1.7%)	2 (6.2%)		
PD-L1/CTLA-4	19 (17%)	7 (22%)		
Other	3 (2.6%)	1 (3.1%)		
WHO status at ICI			0.11	0.3
0	36 (32%)	15 (47%)		
>0	78 (68%)	17 (53%)		
NA	1	0		
Smoking status			0.2	0.5
Never	28 (31%)	5 (19%)		
Smoker	63 (69%)	22 (81%)		

NA	24	5		
Cerebral metastasis	20 (18%)	4 (12%)	0.5	0.6
NA	1	0		
Liver metastasis	34 (30%)	13 (41%)	0.2	0.5
NA	1	0		
Bone metastasis	39 (34%)	8 (25%)	0.3	0.5
NA	1	0		
Lymph node metastasis	75 (66%)	27 (84%)	0.049	0.3
NA	2	0		
Lung metastasis	49 (46%)	9 (29%)	0.088	0.3
NA	9	1		
Pleuro-peritoneal metastasis	47 (41%)	8 (25%)	0.094	0.3
NA	1	0		
Toxicity	40 (35%)	21 (66%)	0.002	0.02
Use of corticosteroids	35 (30%)	14 (44%)	0.2	0.4
Cancer			0.089	0.3
Breast	8 (7.0%)	3 (9.4%)		
Colon	10 (8.7%)	8 (25%)		
Lung	45 (39%)	10 (31%)		
Other	52 (45%)	11 (34%)		

Continuous variables were described by median values and interquartile range (IQR). Categorical variables were described by number of observation and percentages (%).
NA: Not Available; ICI: Immune Checkpoint Inhibitor; PD-1: Programmed cell Death protein 1; PD-L1: Programmed Death-Ligand 1; CTLA-4: Cytotoxic T Lymphocyte–Associated protein 4; WHO: World Health Organization.

Supplementary Table S2: Exome-derived variables in the whole cohort by Recist criteria

Variables		Progresssion N = 115	Response N=32	P-value	Adjusted P-value
TMB status				0.028	0.3
	Low	105 (91%)	24 (75%)		
	High	10 (8.7%)	8 (25%)		
TMB score		4.3 (2.7, 6.6)	4.7 (3.3, 9.2)	0.2	0.3
MSI status				0.2	0.3
	MSS	111 (97%)	29 (91%)		
	MSI	4 (3.5%)	3 (9.4%)		
MSI score		1.3 (0.9, 1.8)	1.3 (1.1, 2.0)	0.3	0.5
TCR clonality		7 (4, 12)	6 (3, 13)	0.7	0.7
BCR clonality		0.00 (0.00, 2.00)	1.00 (0.00, 2.25)	0.2	0.3
	NA	2	0		
Neopeptides		11 (7, 26)	20 (8, 47)	0.12	0.3
	NA	3	0		
Strong neopeptides		2.0 (0.0, 5.0)	2.5 (1.0, 5.2)	0.11	0.3
	NA	3	0		
CNV signature 1		68 (52, 80)	65 (50, 76)	0.7	0.7
	NA	2	0		
CNV signature 2		0.00 (0.00, 0.00)	0.00 (0.00, 0.00)	0.2	0.4
	NA	2	0		
CNV signature 3		13 (10, 20)	13 (10, 19)	0.6	0.7
	NA	2	0		
CNV signature 4		0.00 (0.00, 1.23)	0.00 (0.00, 0.00)	0.017	0.3
	NA	2	0		
CNV signature 5		10 (5, 17)	16 (6, 25)	0.090	0.3
	NA	2	0		
CNV signature 6		0.00 (0.00, 0.00)	0.00 (0.00, 0.00)	0.12	0.3
	NA	2	0		
CNV signature 7		0 (0, 8)	0 (0, 12)	0.6	0.7
	NA	3	0		
TCR Pielou's score		0.98 (0.96, 1.00)	0.99 (0.96, 1.00)	0.7	0.7
	NA	8	2		
BCR Pielou's score		1.00 (0.89, 1.00)	0.96 (0.00, 1.00)	0.2	0.3
	NA	67	12		
TCR Shannon entropy		2.85 (2.14, 3.53)	2.70 (1.65, 3.63)	0.7	0.7
	NA	8	2		
BCR Shannon entropy		1.58 (1.00, 2.27)	1.00 (0.00, 1.92)	0.13	0.3
	NA	67	12		
KRAS				0.12	0.3
	WT	99 (86%)	31 (97%)		
	Mutated	16 (14%)	1 (3.1%)		
STK11				>0.9	>0.9
	WT	108 (94%)	31 (97%)		
	Mutated	7 (6.1%)	1 (3.1%)		

APC			0.6	0.7
WT	111 (97%)	30 (94%)		
Mutated	4 (3.5%)	2 (6.2%)		
RNF43			0.12	0.3
WT	112 (97%)	29 (91%)		
Mutated	3 (2.6%)	3 (9.4%)		
CD274			0.7	0.7
Amplification	8 (7.0%)	1 (3.1%)		
No amplification	107 (93%)	31 (97%)		

Continuous variables were described by median values and interquartile range (IQR). Categorical variables were described by number of observation and percentages (%).
TMB: Tumor Mutational Burden; MSI: Microsatellite Instability; MSS: Microsatellite Stable; TCR: T-Cell Receptor; BCR: B-Cell Receptor; CNV: Copy Number Variant, WT: Wild-Type
